# Refining risk assessment in cardiac sarcoidosis: a role for burden of late gadolinium enhancement and right ventricular function

**DOI:** 10.1186/1532-429X-16-S1-P302

**Published:** 2014-01-16

**Authors:** Gillian Murtagh, Karima Addetia, Amit Patel, Luke Laffin, Francesco Maffessanti, John F Beshai, Roberto Lang, Victor Mor-Avi, Amit R Patel

**Affiliations:** 1 University of Chicago, Chicago, Illinois, USA

## Background

The presence of myocardial late gadolinium enhancement (LGE) in patients with sarcoidosis is a powerful predictor of major adverse events (MAE: death, ventricular tachycardia, or appropriate ICD therapy). In this study, we aim to determine if the burden of LGE, left ventricular (LV) and right ventricular (RV) end-diastolic and end-systolic volume index (EDVi & ESVi) and ejection fraction (EF) can be used to improve risk stratification in patients with cardiac sarcoidosis (CS).

## Methods

**Figure 1:**
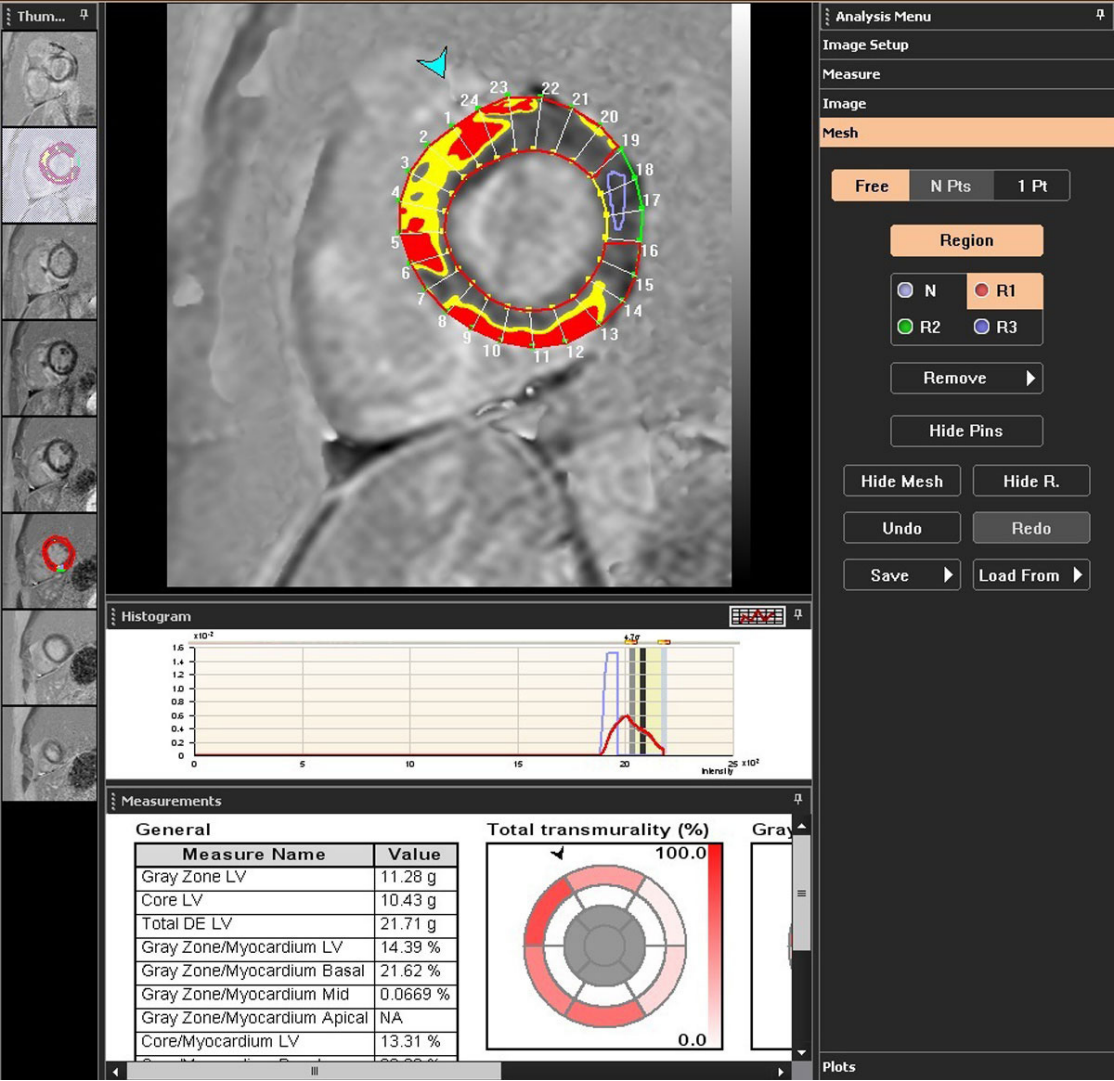
**Diagnosoft Virtue software was used to delineate areas of LGE (defined here as having a signal intensity > 5 standard deviations above that of normal remote myocardium)**.

We identified 25 consecutive subjects with a diagnosis of CS (biopsy-proven extra-cardiac sarcoidosis and presence of LGE on cardiovascular magnetic resonance). Imaging was performed on a 1.5T scanner. Short axis cines (6 mm thickness, 4 mm gap, temporal resolution < 40 ms) spanning the entire LV were acquired to determine LV and RV volumes. Short axis slices were also obtained 10 minutes after contrast (gadodiamide 0.15 mmol/kg) using a phase sensitive inversion recovery reconstruction. LGE was identified on each slice as regions with signal intensity (SI) > 5 standard deviations above the mean SI of normal remote myocardium. The total amount of LGE as a percentage of LV mass (%LGE) was determined using Diagnosoft Virtue software (Figure 1). Medical records were reviewed to identify MAE. Area under the curve (AUC) was determined from receiver-operator characteristics demonstrating the ability of %LGE, LVEDVi, LVESVi, LVEF, RVEDVi, RVESVi, and RVEF to detect MAE. Patients were divided into 2 groups; with MAE and without MAE. Each parameter was evaluated using unpaired T-tests. Continuous variables are presented as mean ± standard deviation.

## Results

The mean age was 58 ± 7 years, 18 (72%) were female. Mean follow up was 39 ± 15 months. Six (24%) of patients had MAE. There was a significant difference in %LGE, RVEF and RVESVi but not LVEF, LVESVi, LVEDVi or RVEDVi between those who had MAE and those who did not (Table 1). The AUC for each of the following parameter's ability to predict MAE was: LVEF 0.54, LVEDVi 0.58, LVESVi 0.62, LGE 0.67, RVEDVi 0.76, RVESVi 0.85, and RVEF 0.56. Notably, in terms of predicting death, the AUC for %LGE, RVEDVi and RVESVi were high at 0.87, 0.86 and 0.89 respectively, while that for LVEF was 0.61, RVEF 0.61, LVEDVi 0.54 and LVESVi 0.55.

**Table 1:** Functional and structural parameters for subjects with and without major adverse events

	MAE +	MAE -	P value
LVEF (%)	59 ± 4	60 ± 4	0.53

LVEDVi (ml/m2)	65 ± 22	27 ± 9	0.53

LVESVi (ml/m2)	27 ± 9	28 ± 7	0.72

RVEF (%)	42 ± 12	54 ± 9	0.01

RVEDVi (ml/m2)	86 ± 22	72 ± 14	0.09

RVESVi (ml/m2)	54 ± 18	34 ± 14	0.007

LGE (%)	20 ± 20	7 ± 5	0.01

## Conclusions

The burden of LGE and RV size and function (not LV size and function) further improve prediction of death and significant ventricular arrhythmia in patients with cardiac sarcoidosis

## Funding

None.

